# Reduced Heart Rate Variability and Altered Cardiac Conduction after Pre-Eclampsia

**DOI:** 10.1371/journal.pone.0138664

**Published:** 2015-09-25

**Authors:** Malia S. Q. Murphy, Geoffrey E. J. Seaborn, Damian P. Redfearn, Graeme N. Smith

**Affiliations:** 1 Department of Biomedical and Molecular Sciences, Queen`s University, Kingston, Ontario, Canada; 2 School of Computing, Queen`s University, Kingston, Ontario, Canada; 3 Department of Cardiology, Kingston General Hospital, Kingston, Ontario, Canada; 4 Department of Obstetrics and Gynecology, Kingston General Hospital, Kingston, Ontario, Canada; University of Adelaide, AUSTRALIA

## Abstract

Pre-eclampsia is a hypertensive disorder of pregnancy that is associated with elevated maternal risk for cardiovascular disease. The aims of this study were to determine the effect of normal pregnancy on postpartum parameters of the electrocardiogram, and furthermore to determine how a history of pre-eclampsia may affect these parameters. Ten-minute high-resolution (1000 Hz) orthogonal Holter electrocardiogram (ECG) recordings were used to measure heart rate variability (HRV). Signal-averaged P-wave and QRS complex durations were determined. Participants included non-pregnant controls, normotensive parous controls and women with a recent history of PE. While reductions in HRV induced by uncomplicated pregnancy returned to non-pregnant levels by 6–8 months postpartum HRV remained reduced in women with a history of PE compared to control groups. In addition, P-Wave and QRS complex durations were prolonged in PE subjects at 6–8 months postpartum compared to control groups. Only QRS duration was independent of differences in blood pressure. These results suggest increased sympathetic cardiac activity, and delayed myocardial conduction in women after PE; alterations consistent with cardiac remodeling and increased risk for arrhythmia. In examining the association between PE and cardiovascular disease, identification of ECG abnormalities soon after pregnancy in women with a history of PE highlights a unique opportunity for early identification and screening in this population before other risk factors become apparent.

## Introduction

Pre-eclampsia (PE) is a common hypertensive disorder of pregnancy that affects 6–10% of pregnancies worldwide. In addition to posing severe maternal and neonatal complications in the peripartum period, PE is associated with increased maternal risk of cardiovascular disease and stroke in later life [1]. The American Heart Association’s *Effectiveness-Based Guidelines for the Prevention of Cardiovascular Disease in Women* now recommends including the development of pregnancy-related complications, including PE, in risk screening practices for heart disease and stroke [2]. In addition, a recent evidence-based review has called for the implementation of cardiovascular risk screening clinics designed to target postpartum women with a history of indicated pregnancy complications [3]. While it remains uncertain whether PE exacerbates previously unrecognized cardiovascular risk factors, or if cardiovascular risk associated with PE is the direct result of the manifestation of the disorder itself, developing an understanding of the early postpartum implications of PE on the maternal cardiovascular system remains important if targeted prevention and screening are to be successful.

It has long been recognized that fluctuations in heart rate can be used to indirectly assess cardiac regulation [4]. In addition to traditional ECG parameters, analysis of HRV is becoming increasingly used as a non-invasive clinical tool for its reliability as an indicator of risk for adverse cardiac events in otherwise normal healthy subjects [5]. Increases in cardiac output as a result of increased blood volume in pregnancy have been shown to influence cardiovascular regulation by way of reduced HRV, blood pressure variability and baroreflex sensitivity [6, 7]. In PE, increases in sympathetic activity and reduced baroreflex sensitivity have been well-described [6–10], although postpartum cardiac electrophysiology and function have been infrequently examined in this population.

As women with PE are at significantly increased risk of heart failure and both atrial and ventricular dysrhythmia in the years after pregnancy [11] assessment of the cardiac state in the earliest times postpartum remains critical in identifying those individuals at greatest risk. The aims of this study were to determine the effects of normal pregnancy on postpartum parameters of autonomic activity and furthermore how a history of PE may affect cardiovascular modulation in the early times postpartum by use of short-term ECG recordings.

## Methods

### Subject identification and follow-up

Approval for this study was obtained from the Queen’s University Health Sciences Research Ethics Board (OBGY-232-12, OBGY-233-12). Written informed consent was obtained from all participants. Fifteen naturally-cycling individuals served as never-pregnant controls. Twenty healthy women experiencing uncomplicated normotensive pregnancies were identified upon routine presentation to low-risk obstetrical clinics and examined in the third trimester, in the early postpartum (5–10 weeks postpartum) and in the late postpartum (6–8 months postpartum).

Due to the variable effects of anti-hypertensive and anti-seizure medications on cardiovascular function it was not feasible to perform measurements in the time leading up to delivery for women with PE. For this reason twenty women with a diagnosis of PE were recruited in the few days following delivery at Kingston General Hospital and consented for follow-up at 6–8 months postpartum. A clinical diagnosis of PE was confirmed by chart review using the newest 2014 guidelines, published in Pregnancy Hypertension [12] (*i*.*e*., ≥140 mmHg systolic blood pressure and/or ≥90 mmHg diastolic blood pressure with new-onset proteinuria or one or more adverse/severe conditions indicated by the new guidelines). Patients with hemolysis, elevated liver enzymes, low platelets (HELLP) were not included in the study. All individuals with a pre-pregnancy history of hypertension, diabetes (including the development of gestational diabetes), renal disease, cardiovascular disease, or who currently were smoking were excluded.

In addition to ECG recordings, information on subject age, parity, heights and weight were collected. An automatic multiple blood pressure recording device (BpTRU; VSM Med-Tech Ltd, Vancouver, Canada) was used to measure the average of five consecutively taken blood pressures.

### Electrocardiography

Experiments were performed in a quiet temperature-controlled room with participants seated in a semi-supine position. All participants were asked to abstain from caffeine intake and over the counter medication use the morning of the study visit. Skin was cleansed with 70% isopropyl alcohol prior to positioning of electrodes in an orthogonal manner. Ten-minute high-resolution (1000 Hz) Holter ECG recordings were collected using a 3 lead SpiderView digital ECG Holter recorder (ELA Medical, Montrouge, FR). Algorithm-based methods filtered for and excluded all non-sinus beats. Validity of exclusion was confirmed by the operator and approximately 5 beats before and after each non-sinus beat were also excluded from analysis. Any R-wave detection errors were corrected for following operator inspection. A summary of ECG parameters assessed in this study are presented in Table 1.

**Table 1:** Summary of ECG parameters assessed.

Parameter	Application
**Time-Domain Variables of HRV**	Time-domain HRV indices mathematically describe the variability in duration between successive RR intervals. Reduced variability of time-domain indices is reflective of mortality risk in a variety of disease states.
Mean RR	Average duration of successive R-R intervals.
SDNN	Standard Deviation of Normal-Normal RR Intervals.
RMSSD	Root Mean Square Successive Difference.
pNN50	Proportion of R-R intervals differing from their directly adjacent R-R intervals >50ms.
**Frequency-Domain Variables of HRV**	Non-linear analysis of R-R interval series using Fast-Fourier Transformation techniques. Frequency-domain indices of HRV provide an evaluation of the contributions of pre-determined frequency ranges to the overall variability in the R-R interval signal.
LF	Low Frequency (0.04–0.15Hz); regarded as a marker of both sympathetic and parasympathetic modulation. Reported in normalized units, LF / (LF + HF).
HF	High Frequency (0.15–0.4 Hz); reflects parasympathetic activity.
LF:HF	A ratio used as a measure of sympathovagal balance.
**P-Wave Duration**	P-wave represents atrial depolarization. Long P wave duration indicates a slowing of electrical conduction throughout the atrium, and may occur in left atrial enlargement.
**QRS Duration**	QRS complex corresponds to ventricular depolarization. Broad QRS complexes indicate aberrant conduction of supraventricular complexes.

### P-wave and QRS duration

Signal-averaged P-wave (SAPW) and QRS (SAQRS) analyses were performed using custom averaging software [13] using the same raw ten-minute high-resolution (1000Hz) Holter ECG recordings used for HRV analysis. As previously described [14], ECG signals were amplified 10 000 times and band-pass filtered between 1Hz and 300 Hz. The lead exhibiting the clearest P-wave or QRS-complex was further filtered between 20 Hz and 50 Hz, and used as a trigger to align subsequent signals for averaging. Averages from orthogonal leads were combined to produce a single vector magnitude display from which the P wave and QRS limits were defined. The analogue data were sampled at 1 kHz with 12-bit resolution and a minimum of 100 beats were used to produce an averaged value of P-wave and QRS-complex duration.

### Statistical analysis

Demographic variables are presented as mean±standard deviation (SD) unless otherwise stated. An unpaired t-test or one-way analysis of variance (ANOVA) with Bonferroni post hoc test was used to compare continuously distributed variables and a χ^2^ comparison was used for categorical measures. GraphPad Prism 5 Software (La Jolla, CA, USA) was used for statistical analyses and comparison within and between subject groups. Normality of data was determined using the D’Agostino and Pearson omnibus normality test, and parametric or non-parametric statistical analysis was completed accordingly. Comparison of normotensive pregnancy control data across experimental time-points were analyzed by matched two-way ANOVA. Comparisons between subject groups, by time-point of measurement were achieved by unpaired one-way ANOVA. Multivariable regression was performed using SPSS software (IBM SPSS Version 22.0, Armonk, New York). Due to the known impact of blood pressure on P-wave and QRS duration, regression models were generated to control for systolic and diastolic blood pressure in the comparison of P-Wave and QRS duration across subject groups. Statistical significance was accepted if the null hypothesis could be rejected at *p*<0.05.

## Results

### Participant Characteristics

Characteristics of the enrolled subjects are summarized in Table 2 **.** Fifteen naturally-cycling NP controls, twenty women with uncomplicated pregnancy and twenty women with a recent history of PE were initially recruited into the study.

**Table 2:** Subject characteristics at time of examination.

	NP n = 15	Uncomplicated Pregnancy n = 20			PE n = 20
	Mid-Cycle	3TM	Early PP	Late PP	Late PP
*Baseline/Obstetrical Characteristics*					
Age (y)	23.5±3.5	29.9±3.72^a^			32.12±7.16^a^
Primiparity, n (%)	-	8 (40)			8 (40)
Pre-pregnancy BMI (kg/m^2^)	22.2±2.7	23.7±3.8	-	-	26.5±8.2
SBP	103.8±6.2	112.4± 6.9^a^	-	-	168.8±17.7^a^ ^,^ ^b^
DBP	68.2±7.6	72.4±7.3	-	-	103.2±8.4^a^ ^,^ ^b^
GA delivery (wks)	-	39.9±1.1	-	-	35.06±3.5^b^
*Postpartum Characteristics*					
BMI (kg/m^2^)	-	-	25.9±3.7	24.5±4.9	29.1±8.0^a^
SBP (mmHg)	-	-	107.2 ±6.9	105.2±8.6	122.4±13.3^a^ ^,^ ^b^
DBP (mmHg)	-	-	68.9±6.9	69.4±7.8	83.9±9.9^a^ ^,^ ^b^

NP, never-pregnant; GA, gestational age; BMI, body mass index; SBP, systolic blood pressure; DBP, diastolic blood pressure; 3TM, third trimester; Early PP, Early postpartum period (5–10 weeks); Late PP, Late postpartum period (6–8 months).

^a^ *p*<0.01 versus never-pregnant

^b^ *p*<0.01 versus time-matched uncomplicated pregnancy.

Women experiencing uncomplicated pregnancy were measured at 37.4±1.4 weeks gestation, and returned for follow-up measurements once in the early postpartum period within 5–10 weeks of delivery (7.37±1.07 weeks) and once in the late postpartum period, 6–8 months postpartum (26.50±3.64 weeks). PE women were assessed once in the late postpartum period, 6–8 months postpartum (26.13±3.64 weeks). PE subjects exhibited statistically elevated systolic and diastolic blood pressures compared to NP and time-matched postpartum controls. Seventeen (85%) postpartum control and eleven (55%) PE women were still breastfeeding in the late postpartum period (*p* = 0.082). Six (30%) parous control and fourteen (70%) PE women had resumed their normal menstrual cycles by that time (*p* = 0.056). In addition, mean RR (average duration of RR intervals) in PE subjects was significantly reduced in the late postpartum period compared to time-matched and NP controls. Although uncomplicated pregnancy was associated with reduced mean RR compared to never pregnant controls, these differences resolved postpartum. No patients were using anti-hypertensive or other prescription medications at the time of follow-up. Preliminary work determined there to be no significant effect of natural hormonal cycling and oral contraceptive use on heart rate variability (data in S1 Appendix). For this reason, data collected during the mid-cycle of naturally-cycling never-pregnant controls were used for comparison to parous subject groups. In acknowledgment of the limited sample sizes postpartum, PE data was not stratified by onset (early/late) or severity (mild/severe) PE.

### Time domain parameters of HRV

**Fig 1:**
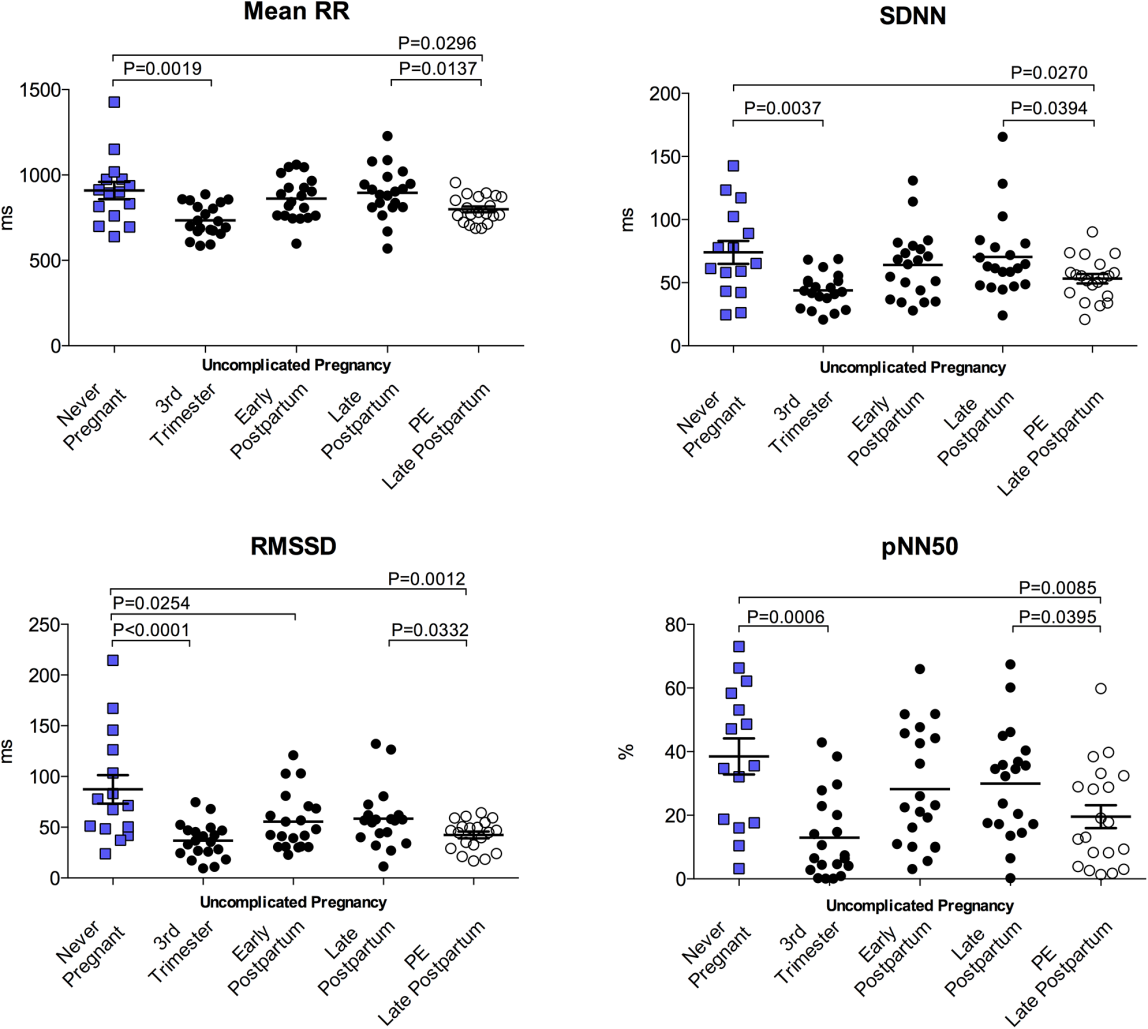
Time domain indices of HRV. Mean RR and time domain indices of HRV were reduced in the 3^rd^ trimester of uncomplicated pregnancy. Time domain parameters of HRV return to never-pregnant levels by the late postpartum (6–8 months), whereas in PE subjects these variables were reduced compared to never-pregnant and time-matched postpartum controls. PP, postpartum.

**Fig 2:**
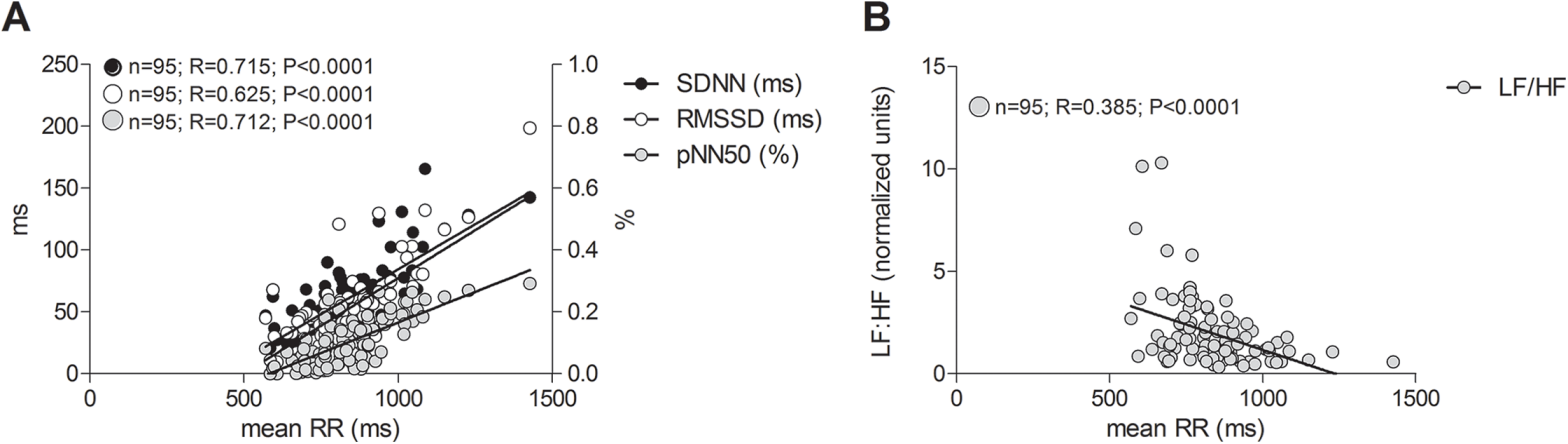
Correlation of heart rate variability parameters to mean RR. All (A) time domain; SDNN, RMSSD, pNN50 and (B) frequency domain; LF/HF parameters were significantly correlated to corresponding mean RR duration in study subjects.

All time-domain indices of HRV were significantly reduced in the third trimester of uncomplicated pregnancy compared to never-pregnant controls. These measures returned to non-pregnant levels by the late postpartum period. In addition, PE subjects exhibited significantly reduced SDNN, RMSSD and pNN50 compared to never-pregnant and time-matched postpartum controls. Time-domain parameters are summarized in Fig 1 **.** Comparison of available paired data from 15 PE subjects at both early and late postpartum timepoints indicated no changes in time-domain parameters across the postpartum period after PE (PE early postpartum vs late postpartum: Mean RR (ms), 851.0±128.0 vs 794.2±86.24; SDNN (ms), 55.35±17.84 vs 50.17±15.28; RMSSD (ms), 49.59±25.09 vs 40.67±15.30; pNN50 (%), 27.16±21.43 vs 19.55±17.67, all *p*>0.05). Time domain parameters of HRV were all highly correlated to mean RR duration Fig 2A.

### Frequency domain parameters of HRV

**Fig 3:**
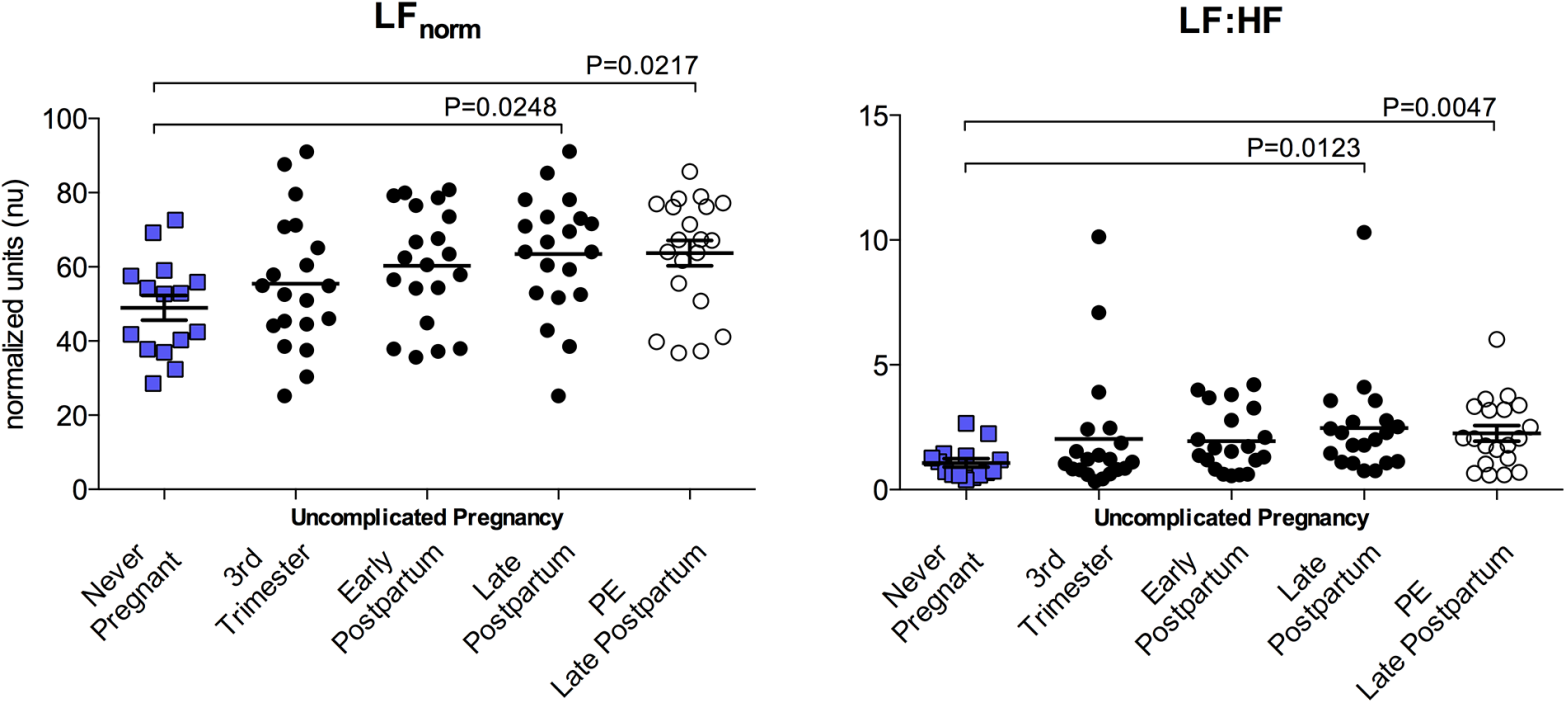
Frequency domain indices of HRV. Frequency domain indices of HRV were not significantly altered by uncomplicated pregnancy. Late postpartum (6–8 months) PE subjects and time-matched controls had significantly increased LF components compared to Never-pregnant controls.

Frequency domain indices were unaltered by normotensive uncomplicated pregnancy. PE subjects exhibited similar degrees of LF_norm_, and LF:HF modulation of heart rate 6–8 months postpartum compared to time-matched postpartum controls, although frequency domain indices from both postpartum groups displayed significant deviations from never-pregnant controls. Frequency domain parameters are summarized in Fig 3. Comparison of available paired data from 15 PE subjects at both early and late postpartum timepoints demonstrated a recovery of autonomic activity similar to that observed after uncomplicated pregnancy (PE early postpartum vs late postpartum: LF_norm_ (nu) 54.29±19.84 vs 65.51±14.80; *p*>0.05). As with time domain parameters, frequency domain parameters of HRV were all highly correlated to mean RR duration (Fig 2B).

### P-wave and QRS Duration

**Fig 4:**
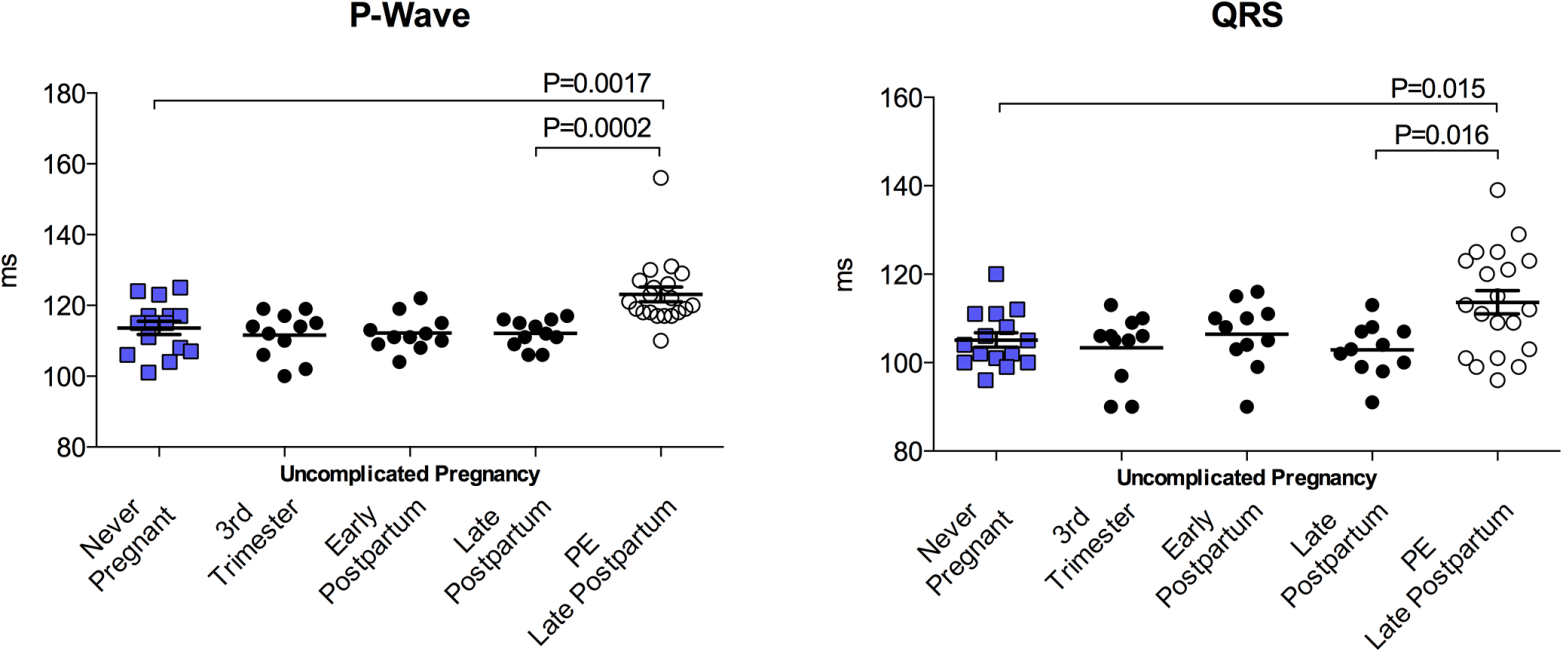
P-Wave and QRS complex duration. Uncomplicated pregnancy, was not associated with alterations in P-Wave or QRS duration compared to never-pregnant controls. Late postpartum (6–8 months), PE subjects exhibited increased duration in both P-wave and QRS complexes.

Not all heart rate recordings were suitable for signal-averaged analysis. For this reason sample sizes for P-Wave and QRS duration data were minimally reduced. P-wave and QRS duration were unaffected by uncomplicated pregnancy. PE subjects presented with significantly longer P-wave and QRS complex durations compared to NP and normotensive time-matched controls; 25% (n = 5) of these were marginally pathological (>20ms). Data are summarized in Fig 4. Available paired data from 11 PE subjects indicated that P-wave and QRS duration remained consistent across the postpartum period (PE early postpartum vs postpartum postpartum: P-Wave (ms), 122.1±6.68 vs 124.4±11.54; QRS (ms), 114.7±12.20 vs 115.0±13.56; both *p*>0.05).

After multiple regression controlling for systolic and diastolic blood pressures, signal averaged P-wave durations at 6–8 months postpartum were not significantly different between PE subjects and never-pregnant and postpartum controls. In brief, using PE as the reference group, never-pregnant subjects had P-wave durations an average of 2.2 ms longer (*p* = 0.743, 95%CI -11.13, 15.48), while women with a history of uncomplicated pregnancy had P-wave durations that were on average 6.1 ms shorter (*p* = 0.363, 95%CI -19.50, 7.29) at 6–8 months postpartum. In contrast, QRS duration remained significantly longer for the PE versus comparison groups after adjustment for blood pressures. Again with PE as the reference group, never-pregnant subjects had QRS durations on average 8.8 ms shorter (*p* = 0.026, 95%CI -16.57, 1.08), and subjects with a history of uncomplicated pregnancy had QRS durations that were on average 10.7 ms shorter (*p* = 0.008, 95%CI -18.50, -2.92) at 6–8 months postpartum.

## Discussion

Using short-term ECG recordings we have demonstrated that women with a recent history of PE exhibit reduced time domain parameters of HRV compared to never-pregnant and time-matched postpartum controls. Examination of P-wave and QRS complex durations in our study cohort further revealed abnormalities of cardiac electrophysiology in PE subjects in the late postpartum period. Of these, only differences in QRS duration appeared to be independent of blood pressure, however. As uncomplicated pregnancy appeared to have no effect on cardiac conduction, prolonged QRS duration 6–8 months postpartum of PE suggests that significant increases in myocardial conduction times may have been present before pregnancy itself. Indeed secondary analysis of paired data at early and late postpartum time points in this group of women indicate that HRV, P-wave and QRS parameters remain stable from the earliest times after pregnancy.

Alterations to cardiovascular variability are likely established as early as six weeks after conception and reflect pregnancy-related increases in heart and respiratory rates [15]. Our findings confirm those of others [6–8, 16] who report variable reductions in time and spectral indices of HRV late in gestation of normotensive pregnancies. Postpartum analyses of cardiovascular variability are few, although our findings of slow postpartum normalization of HRV are in agreement with evidence of persistent alterations to HRV and baraoreflex sensitivity parameters 4 days after uncomplicated pregnancy [17]. Also substantiating our findings, in the same study Walther *et al*. identified that elevated blood pressure and blood pressure variability in PE did not resolve after pregnancy compared to pregnant and non-pregnant controls. 24-hour ambulatory measures of HRV further indicate reduced vagal modulation of heart rate at 3–6 months postpartum in PE subjects [18], and along with our findings suggest a potentiation of cardiovascular risk soon after PE.

Prolonged P-wave duration, QT interval and QT dispersion have been consistently reported in women with PE [19, 20]. Variable effects of steroid and antihypertensive medication often used in this population are considered important determinants of ECG parameters however. In our study observations of persistently increased blood pressure, prolonged P-wave and QRS complexes 5–10 weeks and 6–8 months after PE prompt concern over the severe cardiovascular risks associated with these measures [21–23]. Although differences in P-wave duration were resolved after adjustment for blood pressure in our study, slight increases in blood pressure alone after PE is cause for concern given the impact of even small increases in blood pressure on risk of adverse cardiovascular event s [24].

Reverse remodeling of P-wave duration has been described in some situations, however the presence of significant QRS conduction delay some 6–8 months postpartum speaks to structural atrial remodeling that persists and confers an increased risk of cardiac arrhythmia. Through examination of a large retrospective cohort, the HAD MPS study demonstrated adjusted hazard ratios of 1.56 (95%CI, 1.22–2.00) for not only atrial or ventricular arrhythmia, but also for heart failure a median of 7.8 years postpartum after PE compared to women who did not develop the hypertensive disorder [11]. Risk of arrhythmia or heart failure was particularly increased in women who developed severe PE, 2.00 (95%CI, 1.22–3.20), or PE with preterm delivery 2.31 (95%CI, 1.54–3.48).

Our data is further supported by recent findings of cardiac remodeling and changes in cardiac function postpartum of PE. Left ventricular hypertrophy and concentric remodeling is shown at 1 year postpartum following pre-term development of PE, with increased likelihood of developing asymptomatic stage B heart failure compared to PE acquired at term [25]. As left ventricular dysfunction may persist 13–18 years postpartum beyond the index pregnancy [26], identification of cardiac dysfunction soon after delivery provides an opportunity to identify high-risk individuals before other risk factors become apparent. Although routine screening for non-specific ECG abnormalities in all women with a history of PE may not be a practical approach to reduce CVD burden in this population, it may well be useful when persistent hypertension has been identified [27]. 12 week aerobic exercise and dietary programs are shown to improve retrograde shear rate, intima media thickness, conduit artery flow mediated dilation, and LF:HF specifically in this population, at 6–12 months postpartum [28, 29]. Therefore long-term improvements in cardiac function and risk may be achievable through education and exercise-based intervention.

As a study of relatively small sample size we do caution against extrapolating or generalizing our findings. Indeed, the robustness of multivariable modelling used in this study is limited and so this does not rule out the possibility of interplay between unfavourable biophysical parameters and endothelial dysfunction described in PE subjects in the early postpartum period. Although HRV in women fell to within non-pregnant limits after uncomplicated pregnancy, it was not determined whether these parameters were similar or different from pre-pregnant values for those same women. Similarly, pre-conception, and gestational data for PE women were not compared in this study. It remains unclear why LF:HF ratios were elevated in both postpartum groups compared to the non-pregnant state although young age and slight physique in our never-pregnant controls may have influenced these findings. Given that non-pregnant recordings were obtained from never-pregnant subjects, our data also points to a possible long-term effect of pregnancy itself on sympathovagal balance, although this is merely speculation.

Pre-eclampsia has been likened to a cardiovascular stress test capable of identifying women at risk of hypertension, ischemic heart disease and stroke. We have provided novel evidence of ECG abnormalities in the early postpartum after PE that remain stable some 6–8 months after delivery. In the aims of promoting primary prevention of cardiovascular disease in women, our findings underscore the value of targeted postpartum screening in young women exhibiting pregnancy-related cardiovascular risk indicators [30].

## Supporting Information

S1 Appendix Examination of the effect of the menstrual cycle and monophasic oral contraceptives on HRV measurements. (DOCX)
