# Supplementary material for: Reduced Heart Rate Variability and Altered Cardiac Conduction after Pre-Eclampsia
Source: PLoS One. 2015 Sep 25;10(9):e0138664. doi: 10.1371/journal.pone.0138664 (PMC4583376; doi:10.1371/journal.pone.0138664)
Supplement: S1 Appendix — (DOCX) [file pone.0138664.s001.docx]

**Supplementary Material**

**Examination of the effect of the menstrual cycle and monophasic oral contraceptives on HRV measurements**

**Materials and Methods**

**Subject identification**

The study was approved by the Queen’s University ethics committee. All subjects gave written, informed consent. Thirty (n=30), never-pregnant women were recruited for the study based on volunteer participation from the general student and staff community of Queen’s University, at Kingston, Ontario. Participants were included in the study based on contraceptive use: 1) no hormonal contraceptive use (CTRL) and 2) using monophasic oral contraceptives (OC).

Fifteen CTRL women with self-reported regular menstrual cycles participated in the study, each indicating a normal cycle length ranging between 26 – 30 days. Fifteeen additional subjects were included based on voluntary use of monophasic combination oral contraceptives. A monophasic combination pill type includes the same dose of estrogen ethinyl and progesterone over a 21 day period followed 7 day allotment of placebo pills. Details of combination pills consumed by study participants are given in Table 1. Subjects who had stopped using oral contraceptive drugs within the preceding four months, or who had not yet achieved regular menses since stopping oral contraceptive use were not included in the study. Similarly, OC subjects were included only if they had been adhering to their current prescription for a minimum of 4 months, and who achieved regular menses with their monthly abstention from the prescribed oral contraceptives. Individuals with a body mass index (BMI) greater than 30kg/m^2^, irregular menstrual cycles, history of hypertension, cardiovascular disease, and use of cardiovascular medications, history of smoking, diabetes, or currently using an intrauterine device or other hormonal contraceptive options were excluded from the study.

**Calculation of cycle phase**

Each subject was studied three times over the course of a given menstrual cycle. Cycle phase was calculated based on normal cycle duration, and onset of menses. For CTRL subjects, this included menses, follicular (proliferative) and luteal (secretory) phases. Follicular and luteal phases were defined based on self-reported 26, 28 or 30 day cycles: menses (days 2-4 after spotting), follicular (days 8-12, days 10-14, days12-16 respecitvely), luteal (days 18-22, days 20-24, days 22-28 respectively). These phases were determined by comparing previously published studies with similar phase distinctions and consultation with an OB/GYN clinician. Cycle phase and timing of measurements were further confirmed by tracking the onset of consecutive menses periods over the investigatory period. OC subjects were assessed once during menses (days 2-4 after spotting), and twice over the course of their medicated phases (Early Medicated Phase, Late Medicated Phase) to correspond with the time points of assessment used for CTRL subjects.

**Electrocardiography**

Electrocardiography recordings (ECG) were performed in a quiet temperature-controlled room with the subjects seated in a semi-supine position. All subjects were asked to abstain from caffeine intake and over-the-counter medication use the morning of the study visit. Skin was cleansed with 70% isopropyl alcohol prior to positioning of electrodes in an orthogonal manner. Ten-minute high-resolution (1000Hz) Holter ECG recordings were collected using a 3 lead SpiderView^TM^ digital ECG Holter recorder (ELA Medical, Montrouge, FR) and were analyzed as described in the main text.

**Table S1A. Summary of oral contraceptives used by participants.**

| Oral contraceptive brand name | Ethinyl Estradiol Dose | Progestin Formulation and Dose |
| --- | --- | --- |
| Alesse (n=6) | 0.02mg | 0.10 mg levonorgestrel |
| Yasmin (n=4) | 0.03mg | 3mg drospirenone |
| Yaz (n=2) | 0.02mg | 3mg drospirenone |
| Diane 35 (n=1) | 0.035mg | 2mg cyproterone acetate |
| Marvelon (n=1) | 0.03mg | 0.15mg desogestrel |

**Statistical analysis**

Demographic variables are presented as mean±standard deviation (SD) unless otherwise stated. An unpaired t-test or one-way analysis of variance (ANOVA) with Bonferroni post hoc test was used to compare continuously distributed variables and a χ^2^ comparison was used for categorical measures. GraphPad Prism 5 Software (La Jolla, CA, USA) was used for statistical analyses and comparison within and between subject groups. Normality of data was determined using the D’Agostino and Pearson omnibus normality test, and parametric or non-parametric statistical analysis was completed accordingly. Comparison of normotensive pregnancy control data across experimental time-points were analyzed by matched two-way ANOVA. Comparisons between subject groups, by time-point of measurement were achieved by unpaired one-way ANOVA.

**Results**

A summary of physical characteristics of study participants is provided in Table 2. CTRL and OC participants were similar for age, height, weight and body mass index (BMI). Systolic and diastolic blood pressures were significantly elevated amongst users of oral contraceptives. Mean R-R intervals, time domain parameters, and frequency domain parameters of HRV were similar across the time-frames measured for each study group. In addition, comparison of corresponding phases in CTRL and OC groups demonstrated not significant differences in the ECG parameters assessed. These findings are summarized in Table 3.

**Table S1B. Physical characteristics of study participants**.

|  | CTRL (n=15) | OC (n=15) |
| --- | --- | --- |
| Age, yrs | 23.5±3.50 | 21.9±1.55 |
| Height, cm | 164.7±6.55 | 165.9±4.76 |
| Weight, kg | 60.4±8.48 | 65.1±12.9 |
| BMI, kg/m^2^ | 22.22±2.69 | 23.7±4.72 |
| Systolic Blood Pressure |  |  |
| Phase 1 | 102.2±8.06 | 110.2±5.09^a^ |
| Phase 2 | 103.8±6.25 | 111.3±8.22^a^ |
| Phase 3 | 100.3±5.12 | 110.5±4.16^a^ |
| Diastolic Blood Pressure |  |  |
| Phase 1 | 64.8±5.76 | 74.4±6.83^a^ |
| Phase 2 | 68.2±7.57 | 72.9±10.75 |
| Phase 3 | 65.5±7.09 | 73.9±4.91^a^ |
| Reported Cycle Length, days | 29.3±0.976 | 28±0 |

*CTRL*, no oral contraceptive use; *OC*, monophasic oral contraceptive user; *Phase 1*, menses; *Phase 2*, follicular phase of CTRL, early medicated phase of OC; *Phase 3*, luteal phase of CTRL, late medicated phase of OC. Unpaired t-test, two-tailed comparison. Comparison of blood pressure using matched two-way *ANOVA*, with Bonferroni post-hoc test revealed no changes across cycle phases. ^a^*p*<0.05 versus CTRL.

**Table S1C. HRV by menstrual cycle phase and monophasic oral contraceptive use.**

|  | CTRL (n=15) | | | OC (n=15) | | |
| --- | --- | --- | --- | --- | --- | --- |
| HRV Parameter | Menses | Follicular Phase | Luteal Phase | Un-medicated Phase | Medicated Phase I | Medicated Phase II |
| Mean RR (ms) | 941.9±192.2 | 908.8±197.7 | 904.5±166.4 | 851.8±102.9 | 824.1±89.31 | 860.8±72.31 |
| SDNN (ms) | 78.07±36.29 | 74.02±35.35 | 78.65±38.23 | 61.81±24.09 | 64.04±25.57 | 68.06±23.95 |
| RMSSD (ms) | 80.98±47.00 | 87.32±54.30 | 82.34±50.47 | 61.02±28.66 | 61.94±28.10 | 65.99±33.03 |
| pNN50 (%) | 45.89±21.42 | 38.48±21.93 | 45.92±23.49 | 35.41±21.72 | 35.41±18.03 | 39.73±22.06 |
| LF_norm_ | 417.4±0.1535 | 478.7±0.1338 | 461.7±131.8 | 458.1±173.4 | 498.4±181.9 | 440.4±182.6 |
| LF/HF | 892.9±0.7477 | 1069.0±657.2 | 978.0±546.2 | 109.0±780.0 | 1253.0±854.9 | 980.6±657.8 |

*CTRL*, naturally cycling control; *OC*, monophasic oral contraceptive user; *mean RR*, average time between successive R-R intervals; *SDNN*, standard deviation of normal-normal R-R intervals; *RMSSD*, root mean square of the difference between successive R-R intervals; *pNN50*, proportion of R-R intervals differing from directly adjacent R-R intervals >50ms; *LF*, low frequency (0.04-0.15Hz); *HF,* high frequency (0.15-0.4Hz). Data presented as mean±SEM. Comparisons were made by two-way *ANOVA*, with Bonferroni post-hoc test.
